# Non-Pharmacological Treatments for Insulin Resistance: Effective Intervention of Plant-Based Diets—A Critical Review

**DOI:** 10.3390/nu14071400

**Published:** 2022-03-27

**Authors:** Michalina Banaszak, Ilona Górna, Juliusz Przysławski

**Affiliations:** 1 Faculty of Medical Sciences, Poznan University of Medical Sciences, 60-812 Poznan, Poland; mi.banaszak97@gmail.com; 2 Department of Bromatology, Poznan University of Medical Sciences, 60-806 Poznan, Poland; jotespe@ump.edu.pl

**Keywords:** vegetarian diet, vegan diet, insulin resistance, insulin sensitivity

## Abstract

Plant-based diets are becoming increasingly popular. Vegetarian diets are better for the environment and exhibit health benefits. A correctly balanced plant-based diet is appropriate at every stage of life. Compared to omnivores, vegetarians consume more fruits and vegetables, more fibre, vitamins C and E, magnesium and less saturated fats. In general, they have better nutrition knowledge, and they are slimmer, healthier and live longer than omnivores. It also seems that following a plant-based diet prevents the onset of chronic diseases such as cardiovascular diseases, hypertension, type 2 diabetes, obesity and some cancers. Food intake has a key influence on insulin resistance. Consumption of calorie-rich and highly processed foods, meats and sweetened beverages is a characteristic element of Western diets. They promote and elevate insulin resistance and type 2 diabetes. In contrast, intake of pulses and exclusion of meats as well as animal products bring significant benefits to vegetarian diets. According to studies, vegetarians and vegans have better blood parameters, including better glucose, insulin, total cholesterol, and LDL cholesterol levels. Their homeostatic model assessment for insulin resistance (HOMA-IR) test results are also better. More plant-based foods and fewer animal foods in a diet result in lower insulin resistance and a lower risk of prediabetes and type 2 diabetes. The aim of the study was to investigate the effect of plant-based diets on insulin resistance. In this review, we focused on presenting the positive effects of vegetarian and vegan diets on insulin resistance while showing possible clinical applications of plant-based diets in the treatment and prevention of modern-age diseases. Current and reliable publications meeting the requirements of Evidence-Based Medicine (EBM) were taken into account in this review.

## 1. Introduction

Insulin resistance (IR) is a pathological condition in which cells fail to respond normally to insulin. As a result, glucose remains in the bloodstream. It may be defined as a state where normal or elevated insulin levels result in a poor biological response. Insulin resistance is often accompanied by hyperinsulinemia, a condition where pancreatic β-cells release excessive amounts of insulin to maintain normal glycaemia [1,2,3]. Unfortunately, over time, the pancreas is unable to secrete adequate amounts of the hormone. This leads to the development of type 2 diabetes. Apart from diabetes, IR is also associated with the development of metabolic syndrome and cardiovascular diseases [4]. It is estimated that insulin resistance precedes the development of diabetes type 2 by 10 to 15 years [5].

The exact causes of insulin resistance have not yet been fully understood. Increased intramuscular fat and fatty acid metabolites are amongst the suspected risk factors. Excess body weight is considered to be the main cause of insulin resistance. It was suggested that IR starts in myocytes due to immune-mediated inflammatory changes and an excess of free fatty acids resulting in ectopic lipid deposition. In the muscles, the process of glucose uptake is disturbed, so that excess glucose returns to the liver, where it increases lipogenesis and the levels of circulating free fatty acids. Increased levels of free fatty acids exacerbate insulin resistance. There are also genetic causes of insulin resistance, including genetic diseases (myotonic dystrophy, Rabson–Mendenhall syndrome, Werner syndrome) and abnormalities in the function of insulin antibodies and insulin receptors. The gold standard for diagnosing insulin resistance is the hyperinsulinemic-euglycemic clamp. This method involves the continuous infusion of insulin at high speed to inhibit the production of glucose by the liver. Simultaneously, 20% dextrose solution is administered at different rates to reduce blood glucose levels in the euglycemic range. Blood glucose is monitored frequently during the test. The amount of glucose required to achieve normoglycemia reflects the excretion of exogenous glucose required to compensate for hyperinsulinemia. However, as this technique is labour-intensive and costly, indices are used to quantify IR. The homeostatic model assessment for insulin resistance (HOMA-IR) is the prevalent method. It is calculated by multiplying fasting insulin and fasting glucose levels and then dividing that figure by 22.5 [4,5,6].

The primary non-pharmacological treatment for insulin resistance should entail lifestyle approaches. Dietary changes should include a modification of eating habits, reduction in energy intake (for excessive body weight) and avoidance of carbohydrates that excessively stimulate insulin secretion, known as high glycaemic index carbohydrates and high glycaemic load carbohydrates. Regular exercise improves muscle insulin sensitivity [1,5,7,8].

The aim of this review was to show that diets that emphasise whole grains, vegetables, fruits and legumes and exclude animal products are able to reduce insulin resistance and improve tissue insulin sensitivity.

## 2. Materials and Methods

**Figure 1:**
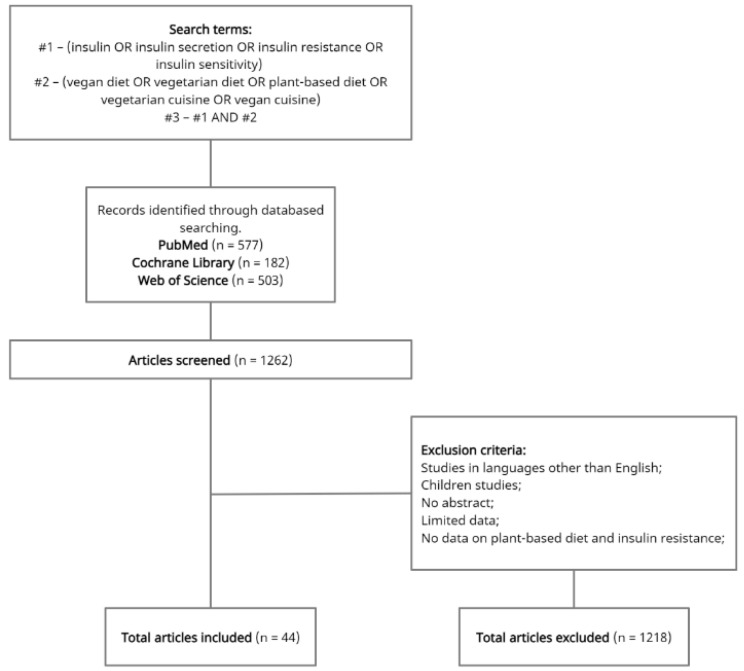
Full search strategy.

Forty-four publications from the PubMed database, the Web of Science and the Cochrane Library were used for this literature review. The following inclusion criteria were applied for the review: papers in English and only research conducted on adults (over the age of 18). The following keywords were used to search for papers: ‘insulin’, ‘insulin secretion’, ‘insulin resistance’, ‘insulin sensitivity’, ‘vegan diet’, ‘vegetarian diet’, ‘plant-based diet’, ‘vegetarian cuisine’, ‘vegan cuisine’. The literature included comparative studies, cross-sectional studies and randomised controlled trials (Figure 1).

## 3. Plant-Based Diets

People are showing increasing interest in plant-based diets. Ethical, environmental and social considerations are some of the primary reasons for changing to a plant-based diet. Health issues are also playing an increasingly significant role [9]. Compared to traditional diets, those based on plants are more environmentally friendly. They use fewer natural resources and cause less pollution [10].

A vegetarian diet involves abstaining from meat, poultry, fish and seafood [11]. There are different types of vegetarian diets. Lacto-vegetarians consume dairy products, ovo-vegetarians consume eggs, whereas lacto-ovo-vegetarians consume eggs and dairy products [12]. On the other hand, vegans abstain from all animal products [13]. Such a diet consists exclusively of plant foods such as cereals, vegetables, fruits, legumes, nuts, seeds and vegetable oils [10,14,15,16]. The most prevalent types of plant-based diets are shown in Table 1.

There is also a semi-vegetarian diet (flexitarian). This eating pattern limits the consumption of meat or fish to a few portions per week. However, it is not considered vegetarian per se [17].

According to the Academy of Nutrition and Dietetics, well-balanced vegetarian diets, including vegan diets, are suitable at all stages of life, including during pregnancy, lactation, infancy, childhood, adolescence and old age. Such direst is also suitable for athletes [10].

Generally speaking, numerous studies indicate that vegetarians are more aware of the health consequences of poor nutrition that they are slimmer and healthier than people who do not follow such restrictions [11]. Compared to non-vegetarian diets, a plant-based diet may prevent the onset of chronic diseases such as cardiovascular diseases, hypertension, type 2 diabetes, obesity and lower overall cancer risk, especially colorectal cancer, breast cancer and prostate cancer [10]. According to research, for vegans, the risk of hypertension is reduced by 75%, the risk of type 2 diabetes is reduced by 47–78% and the risk of cancer is reduced by 14% [18]. Furthermore, plant-based diets are associated with reduced mortality rates for humans [19,20].

Unfortunately, not every diet based on fruit and vegetables is healthy. A poorly balanced vegetarian diet can be just as harmful as an unbalanced traditional diet. Plant-based diets can be deficient in B vitamins (especially B12), iron, calcium, zinc, omega 3 fatty acids and protein. It is, therefore very important to consume fortified foods and dietary supplements [15,17]. There is some concern as to whether a vegan diet can meet the protein requirements of the human body [21]. A well-balanced vegan diet provides all essential amino acids and an adequate amount of total protein without the need to supplement the diet with special foods [10].

The number of people around the world who follow a vegetarian diet is not precisely known. However, every year that number is growing. It is estimated that there may be 7.3 million vegetarians in the USA, 3.6 million in the UK and 30,000 in Portugal [17]. According to estimates, vegans account for as many as 46% of vegetarians [10].

## 4. Impact of a Vegetarian Diet on Insulin Resistance

The foods we eat have a key impact on insulin resistance. This is especially true for the elderly and those who are not very physically active. The increase in the consumption of highly processed, calorie-rich foods such as fast foods, meats and meat products, refined cereal products and soft drinks is estimated to play an important role in the worldwide growth of type 2 diabetes [22]. Balanced and well-composed diets can play a significant role in reducing insulin resistance and the risk of type 2 diabetes (Table 2) [23].

Those following vegetarian diets consume more monounsaturated fatty acids (MUFA), more polyunsaturated fatty acids (PUFA) and less saturated fatty acids (SFA) as compared to non-vegetarians [42]. Saturated fatty acids and palmitic acid, in particular, interfere with insulin signalling in muscle cells due to the accumulation of free fatty acid intermediates, ceramides and diacylglycerol [43,44]. Diets that include animal products contain much more SFAs than plant-based diets. This means that vegetarians and vegans have less insulin resistance [41]. In addition, replacing SFAs in a diet with MUFAs and PUFAs has anti-inflammatory effects and improves insulin sensitivity [45].

Studies have shown that legumes, which are an important source of protein in a vegetarian diet, reduce insulin resistance, which is associated with protection against the onset of metabolic syndrome [24,25,46,47]. Pittaway et al. [26] demonstrated an improvement in tested blood parameters after 12 weeks of consuming chickpeas (minimum 728 g per week). In addition to a reduction in total cholesterol, there was a 0.75 μIU/mL decrease in fasting insulin (*p* = 0.045), and the HOMA-IR insulin resistance index fell by 0.21 compared with the start of the study (*p* = 0.01).

Animal products, in particular red meat, show a tendency to elevate insulin resistance [27,28,48,49,50,51]. A study by Tucker et al. [27] on a group of 292 females without diabetes found that women who consumed large and moderate amounts of meat had significantly higher HOMA-IR insulin resistance index scores than those with a lower meat intake (F = 7.4; *p* = 0.0070). They put forward the idea that meat consumption could lead to insulin resistance. This is associated with a reduced risk of type 2 diabetes for vegetarians compared to omnivores.

Numerous studies show a positive effect of a vegetarian diet on insulin resistance compared to traditional, non-vegetarian diets [29,30,31,32,33,34,35,36,37,38].

In their study, Kahleova et al. [35] compared a vegetarian diet (∼60% energy from carbohydrates, 15% proteins and 25% fats) with a conventional diabetic diet (50% energy from carbohydrates, 20% proteins and <30% fats). A group of 74 patients with type 2 diabetes was randomly assigned to 2 groups: an experimental group (*n* = 37) that followed a vegetarian diet and a control group (*n* = 37) that followed a conventional diabetic diet. The results from the experimental group showed that as many as 43% of those on a vegetarian diet were able to reduce the doses of their diabetes medication (compared with 5% in the control group; *p* < 0.001). Bodyweight also decreased more for those in the experimental group than in the control group (−6.2 kg (95% CI −6.6 to −5.3) vs. −3.2 kg (95% CI −3.7 to −2.5)). Furthermore, there was a significant increase in insulin sensitivity in the experimental group (30%; 95% CI 24.5–39) compared to the control group (20%; 95% CI 14–25) (*p* = 0.04). A vegetarian diet was also shown to reduce visceral adipose tissue volume and positively affect adiponectin and oxidative stress markers. The results indicate that a vegetarian diet alone or in combination with exercise is more effective in reducing insulin resistance than a conventional diabetic diet.

Metabolic abnormalities are a factor that can trigger age-related diseases. This may be more intense for obese individuals. With that in mind, Valachovicová et al. [36] evaluated insulin resistance in relation to diet. Fasting glucose and fasting insulin concentrations, as well as insulin resistance (HOMA) values, were analysed in normal-weight adults. The group included 95 vegetarians and 107 non-vegetarians on a Western diet. The results were significantly lower in the vegetarian group (glucose concentration 4.47 ± 0.05 vs. 4.71 ± 0.07 mmol/L; insulin concentration 4.96 ± 0.23 vs. 7.32 ± 0.41 mU/L; HOMA-IR index 0.99 ± 0.05 vs. 1.59 ± 0.10). Vegetarians were significantly more likely to consume whole grain products, pulses, oat and barley products, as well as fruit and vegetables. Studies show the beneficial effects of long-term use of a plant-based diet in the prevention of metabolic syndrome, diabetes and cardiovascular disease.

On the other hand, Chiang et al. [37] found that vegetarianism was associated with better lipid profiles, insulin resistance and lower risks of metabolic syndrome. The study comprised 391 female vegetarians (80% lacto-vegetarians) and 315 non-vegetarian Buddhist women from Taiwan. The vegetarians had a lower BMI (22.9 ± 2.7 vs. 23.8 ± 3.2 kg/m^2^), smaller waist circumference (72.9 ± 6.9 vs. 75.3 ± 7.6 cm), lower fasting glucose (4.98 ± 0.89 vs. 5.15 ± 0.97 mmol/L) and fasting insulin (41.67 ± 37.50 vs. 52.09 ± 41.67 pmol/L) levels, lower total cholesterol (4.68 ± 0.83 vs. 5.21 ± 0.90 mmol/L) and lower LDL cholesterol (3.01 ± 0.74 vs. 3.43 ± 0.81 mmol/L) compared to non-vegetarians (*p* < 0.001). These vegetarians also had better insulin sensitivity, which was associated with lower HOMA levels (vegetarians 1.3 ± 1.2 vs. non-vegetarians 1.7 ± 1.5; *p* < 0.001). According to the study, a lacto-vegetarian diet was associated with reduced insulin resistance and risk of metabolic syndrome.

Vučić Lovrenčić et al. [38] reached similar conclusions. Female vegetarians had significantly lower fasting insulin levels (38.4 (35.1–46.4) vs. 49.9 (37.7–63.8) pmol/L) and lower HOMA index values (0.80 (0.75–0.95) vs. 1.10 (0.80–1.30)) than omnivorous women (*p* = 0.02) Additionally, pancreatic β-cell function as estimated by HOMA2 (HOMA2%B) was significantly higher in subjects on a plant-based diet than in those consuming meat products (115.5 ± 42.9 vs. 91.0 ± 35.0, *p* = 0.04), indicating that a vegetarian diet had a positive effect on improving β-cell function.

Ellsworth et al. [39] focused on verifying whether lifestyle changes would improve IR in individuals with different severities of metabolic dysfunction. Participants with type 2 diabetes, ischaemic heart disease or at risk from these diseases were allocated to two groups: an intensive non-randomised programme with a strict vegetarian diet (90 participants in the study group, 90 in the control group) or a moderately randomised trial on a Mediterranean diet (89 participants in the study group, 58 in the control group). The research carried out over one year showed that both interventions resulted in weight loss (−8.9% (95% CI, −10.3 to −7.4)—intensive programme; −2.8% (95% CI, −3.8 to −1.9)—moderate programme; *p* < 0.001). Insulin resistance was measured using the Lipoprotein IR Index (LPIR) score. LPIR are six lipoprotein parameters that exhibit the strongest association with insulin resistance. This index may be useful for identifying patients at risk of developing type 2 diabetes and ischaemic heart disease [52]. Insulin resistance significantly decreased for subjects in both groups (LPIR −13.3% (95% CI, −18.2 to −8.3)—intensive; −8.8% (95% CI, −12.9 to −4.7)—moderate; *p* < 0.01). Based on the results, one may conclude that both a strict change to a vegetarian diet as well as a moderate change to a Mediterranean diet is effective in improving IR as defined by LPIR [39].

On the other hand, Garousi et al. [40] compared the effects of a lacto-vegetarian diet with a standard weight loss diet in a group of 75 adults with nonalcoholic fatty liver disease (NAFLD). The nutritional make-up for both diets was similar: 50–55% carbohydrates, 15–20% protein and 25–30% fats. After 3 months of research, alanine aminotransferase (ALT) levels were significantly lower for the lacto-ovo-vegetarian group (−21.32 ± 19.77 vs. −10.15 ± 20.30 IU/L; *p* = 0.04) as compared with the standard weight loss diet. In addition to improving liver function, the lacto-vegetarian diet resulted in significant reductions in body weight, BMI, waist circumference and lipid profile parameters. Additionally, compared to the group on a weight loss diet, the lacto-vegetarian diet resulted in a significant reduction in insulin (−4.94 ± 5.40 vs. +0.81 ± 8.35 μU/mL; *p* = 0.006) and lower HOMA-IR index values (−1.62 ± 1.48 vs. +0.02 ± 2.14; *p* < 0.001). According to research, following a lacto-vegetarian diet had a more beneficial effect on improving the health of NAFLD patients than following a standard reduction diet.

The association of insulin resistance, prediabetes and type 2 diabetes with vegan and vegetarian diets was also analysed. Chen et al. [41] carried out research on a group of 6798 subjects. They showed that more plant-based foods and fewer animal products in a diet result in lower insulin resistance and a lower risk of prediabetes and type 2 diabetes. This suggests that plant-based products may reduce the risk of insulin resistance and its negative effects.

## 5. Impact of a Vegan Diet on Insulin Resistance

Compared to other vegetarian diets, vegans generally consume less saturated fat, less cholesterol and more fibre. Higher intakes of antioxidants and magnesium also elevate insulin sensitivity (Table 3) [53].

The composition of dietary proteins influences glucagon and insulin activity in the body [64], which in turn affects body composition and insulin resistance [65]. It was found that a high intake of branched-chain amino acids (leucine, isoleucine and valine) can increase insulin resistance. Kahleova et al. [54] conducted a study to determine the effects of plant protein on body weight, body composition and insulin resistance in overweight individuals. A group of 75 participants with excessive body weight was randomly assigned to a group on a plant-based diet (*n* = 38) or following a control diet (*n* = 37). The vegan group achieved significant reductions in body weight (−6.5 kg (95% CI −8.9 to −4.1 kg); *p* < 0.001), fat mass (−4.3 kg (95% CI −5.4 to −3.2 kg); *p* < 0.001) and HOMA-IR (treatment effect −1.0 (95% CI −1.2 to −0.8); *p* = 0.004). Interestingly, it was noted that a decrease in fat mass was associated with an increase in plant protein intake and a decrease in animal protein intake (r = −0.30, *p* = 0.011; r = +0.39, *p* = 0.001, respectively). Proportionally reduced dietary leucine intake was positively correlated with decreased fat mass (r = +0.40; *p* < 0.001), and proportionally reduced histidine intake was associated with decreased insulin resistance (r = +0.38; *p* = 0.003). To conclude, plant protein as a component of a plant-based diet was associated with improved body composition, reduced body weight and reduced insulin resistance.

The positive effects of a low-fat vegan diet on insulin resistance and insulin sensitivity index (PREDIM) were shown by numerous studies [55,56,57,58,59,60]. Kahleova et al. [61] used a randomised controlled trial to examine the effects of a low-fat vegan diet on body weight, insulin resistance as well as hepatic and intracellular lipid levels in overweight adults. The study group comprised 244 people, 122 of whom were allocated to an intervention group on a low-fat vegan diet (approximately 75% energy from carbohydrates, 15% from proteins and 10% from fats), with the remaining 122 eating as before during the 16-week study period. Based on the results, participants in the intervention group recorded a weight reduction of 5.9 kg (95% CI, 5.0–6.7 kg; *p* < 0.001). The HOMA insulin resistance index was also lower (−1.3; 95% CI, −2.2 to −0.3; *p* < 0.001) and the predicted insulin sensitivity index (PREDIM) increased (0.9; 95% CI, 0.5–1.2; *p* < 0.001) for the vegan group. The low-fat vegan diet also had a positive effect on lipid levels, with hepatic cell lipid amounts down by 34.4% and intracellular lipids down by 10.4%. The above variables did not change significantly for the control group. The results confirm the positive effects of a plant-based diet on both body weight, insulin resistance and body lipid levels.

Barnard et al. [62] carried out a similar study. A group of 64 postmenopausal women with excessive body weight were randomly assigned to a group on a low-fat vegan diet (10% energy from fats, 15% from protein, 75% from carbohydrates) or a control diet (fat 30%, protein about 15%, carbohydrate 55%). An analysis of these results showed that mean body weight in the intervention group decreased by 5.8 ± 3.2 kg as compared with 3.8 ± 2.8 kg in the control group (*p* = 0.012). On the other hand, the insulin sensitivity index, calculated using a quotient of fasting blood glucose and fasting insulin, mean blood glucose and mean insulin concentration, increased from 4.6 ± 2.9 to 5.7 ± 3.9 (*p* = 0.017) in the intervention group; however, differences between groups were not significant (*p* = 0.17). Studies have shown that despite not specifying recommended portion sizes or amounts of food, women switching to a low-fat vegan diet lost significant amounts of weight.

Śliż et al. [63] compared a vegan diet (*n* = 44) to an omnivorous diet (*n* = 54) among Polish athletes. The vegan group had significantly better HOMA-IR (0.96 ± 0.49 vs. 1.17 ± 0.44; *p* < 0.01), fasting glucose (4.3 ± 0.6 vs. 4.8 ± 0.5 mmol/L; *p* = 0.01) and C-peptide (1.2 ± 0.4 vs. 1.5 ± 0.35 ng/mL; *p* < 0.01) results than the group consuming animal products. Vegan athletes who consumed sufficient macronutrients with their diet had better insulin sensitivity and lower cholesterol levels than omnivorous athletes. However, the content of dietary protein, EPA and DHA may have been insufficient, and supplementation should be considered for vegan athletes.

## 6. Discussion

Research indicates that people decide to change to a plant-based diet mainly for health reasons, then for satisfaction and well-being. The environmental and ethical factors were reported less frequently [66,67]. In addition to many positive effects on human health, plant diets can also show negative features. It was suggested that plant-based diets are suitable for people at all stages of life, as long as they are well-balanced and planned [68]. One of the main objections to using plant-based diets is that they are deficient in protein. Research shows that currently, there are no significant differences in covering protein requirements, provided that plant sources of protein are used: legumes, soy products, nuts and seeds [10,67,69,70]. The nutritional value of legume protein is slightly lower than that of meat. Due to the limiting amino acids in plant protein sources, it is necessary to combine the legumes with cereal products, which supplement the missing amino acids [71,72]. Incorrect selection of plant foods may result in deficiencies of micronutrients, in particular B vitamins (B2 and B12), vitamin D, iron, zinc and calcium. Vitamin B12 is present mainly in animal products (liver, meat, milk, dairy products, eggs); therefore, it is essential to choose products enriched with this vitamin or take dietary supplements [68,73]. It is important to seek health and dietary advice based on scientific evidence [74].

Some people cannot decide to exclude animal products from the diet completely, and therefore they limit their consumption [68]. The maintenance of plant-based diet habits is influenced by many factors, including personal factors, friends and family and the availability of vegetarian and vegan products [68,75]. Questionnaire research indicates that the main barrier to changing eating habits is the pleasure of eating meat and the difficulty in giving up eating it [67,76]. For many people, the difficulty adapting plant diets is the complicated process of preparing meals, the availability of ready-made products and dishes, high prices, lack of variety and unpalatable meals [67,77,78,79,80]. Research shows that replacing animal proteins with legumes is difficult. The respondents indicate that dishes based on legumes are unattractive and unpalatable, which is a severe barrier to plant-based diets [71,81,82,83,84].

In addition, vegetarians can experience discrimination and social constraints. Individuals may feel rejected and judged by other family members due to plant-based diets. Moreover, established eating habits and attitudes constitute a barrier to changing the diet to a plant-based, mainly for the elderly [67,68].

The main limitations of our study include the extensive literature on plant-based diets. Unfortunately, among many articles, few of them focused on the impact of plant-based diets on insulin resistance, which makes the presented results favourable and unambiguous. We did not find any study showing a negative effect of plant-based diets on insulin resistance. Many of the 44 references used for the review were comparative studies. Few randomised controlled trials limit the possibility of unequivocally establishing the effect of plant-based diets on insulin resistance. We believe that more research of this type is needed, setting future research directions. Thanks to the PRISMA diagram, we were able to conduct the above literature review. It made our work easier and systematised the analysis of the topic of the influence of plant-based diets on insulin resistance.

## 7. Conclusions

Vegetarian diets show beneficial effects not only on insulin resistance but also on other health parameters, including body weight, body fat, BMI and lipid profile parameters. Meat-free diets are suitable for everyone, regardless of age or health. Unfortunately, improperly balanced plant-based diets may carry a risk of nutritional deficiencies, in particular deficiencies in protein, B vitamins, iron, zinc and omega 3 fatty acids.

This review has such limitations as studies in languages other than English, children studies, no abstract, limited data and no data on plant-based diet and insulin resistance. However, more research is needed to determine whether plant-based diets can be used to treat diseases.

Based on available research, it may be concluded that vegetarian diets deliver good results, that more plant-based foods and fewer animal products in a diet result in lower insulin resistance and a lower risk of prediabetes and type 2 diabetes.

## Figures and Tables

**Table 1:** Characteristic of plant-based diets.

Dietary Approach	Type of Diet	Characteristics
Plant-based diet	Vegetarian	Does not contain meat, fish or seafood. Contains fruit, vegetables, whole grains, pulses, nuts and seeds. May or may not include egg and/or dairy products.
	Lacto-ovo-vegetarian	Contains eggs and dairy products.
	Lacto-vegetarian	Includes dairy products but excludes eggs.
	Ovo-vegetarian	Includes eggs but excludes dairy products.
	Vegan	Does not contain any animal products. May exclude honey.
	Raw vegan	Includes uncooked vegetables, fruits, nuts, seeds, legumes/beans and whole grains. The amount of uncooked food varies from 75% to 100%.

**Table 2:** The influence of a vegetarian diet on insulin resistance.

Study	Year	Country	Cohort	Analysed Groups	Time of Intervention	Results
Hosseinpour-Niazi et al. [24]	2015	Iran	31 participants (24 women and 7 men; age: 58.1 ± 6.0 years, with type 2 diabetes)	Legume-based Therapeutic Lifestyle Change (TLC) diet Control diet (legume-free TLC diet)	8 weeks	Decreased fasting blood glucose (*p* = 0.04), fasting insulin (*p* = 0.04), triglyceride concentrations (*p* = 0.04) and low-density lipoprotein cholesterol (*p* = 0.02) for TLC group. No change in body mass index (BMI), waist circumference.
Jenkins et al. [25]	2012	Canada	121 participants with type 2 diabetes (60 women, 61 men, aged 59.5 ± 1	(1) Low-GI diet (diet rich in legume) (2) High wheat fiber diet (diet rich in high wheat fiber foods)	3 months	Decreased HbA1c (*p* < 0.001), body weight) (*p* = 0.002), fasting glucose level (*p* = 0.001), systolic BP (*p* < 0.001), diastolic BP (*p* < 0.001), heart rate (*p* < 0.001), absolute CHD risk (10 years) (*p* = 0.003) in both groups.
Pittaway et al. [26]	2008	Australia	45 participants (13 premenopausal women, 19 postmenopausal women, 13 men; age: 52.2 ± 6.1 years)	A diet consisting of a minimum of 728 g of chickpeas per week as part of traditional diet for 12 weeks (chickpea phase), followed by 4 weeks of the traditional diet without chickpeas (usual phase).	20 weeks	Significant decrease in mean serum total cholesterol of 7.7 mg/dL (*p* = 0.002), LDL cholesterol of 7.3 mg/dL (*p* = 0.01), fasting insulin of 0.75 IU/mL (*p* = 0.045) and in HOMA-IR of 0.21 (*p* = 0.01).
Tucker et al. [27]	2015	USA	292 participants (nondiabetic women; age: 40.3 ± 3.1 years)	3 groups: woman with low meat intake (*n* = 73), moderate meat intake (*n* = 164) and high meat intake (*n* = 73)	7 days	Significantly higher HOMA scores in groups with high and moderate meat consumption (*p* = 0.007).
Ley et al. [28]	2014	USA	3690 participants (nondiabetic women from Nurses’ Health Study; age 30–55 years)	-	-	Higher red meat consumption was associated with higher plasma CRP, ferritin, fasting insulin, and HbA1c, and lower adiponectin (*p* ≤ 0.03 for all). Substituting a serving of total red meat intake with alternative protein food showed improvement in lowering CRP, ferritin, HbA1c and fasting insulin levels(*p* ≤ 0.02 for all).
Cui et al. [29]	2019	China	558 participants (healthy men and women, age 32–34 years old)	279 vegetarians (73 vegans, 206 lacto-ovo-vegetarians) and 279 omnivores	3 months	Vegan diet and lacto-ovo-vegetarian diet were negatively correlated with HOMA-IR after adjusting for BMI.
Kim et al. [30]	2015	Korea	102 participants (postmenopausal women, age of 47 to 85 years old)	54 vegetarian women and 48 non-vegetarian women	-	Significantly lower body weight (*p* < 0.01), body mass index (*p* < 0.001), % of body fat (*p* < 0.001), serum levels of leptin (*p* < 0.05), glucose (*p* < 0.001), insulin (*p* < 0.01) and HOMA-IR (*p* < 0.01) in the vegetarians group.
Yang et al. [31]	2012	China	295 participants (men aged 21–76 years)	169 lacto-vegetarians 126 omnivores	-	Remarkably lower body mass index (*p* = 0.049), triglyceride level (*p* = 0.016), total cholesterol (*p* < 0.001), LDL cholesterol (*p* < 0.001) and fasting blood glucose (*p* < 0.001) in lacto-vegetarians group. Higher homeostasis model assessment β cell function (*p* < 0.001) and insulin secretion index (*p* = 0.048) in lacto-vegetarians.
Gammon et al. [32]	2012	New Zealand	124 participants (women at least 20 years old)	90 non-vegetarians 34 vegetarians	-	Increased body mass index, waist circumference and HOMA2-IR levels in non-vegetarians group. Higher serum vitamin B12 levels in non-vegetarians (*p* < 0.001).
Hung et al. [33]	2006	Taiwan	98 participants (healthy women, age 31–45 years old)	49 lactovegetarians 49 omnivores	-	Significantly lower levels of fasting insulin (*p* < 0.001), plasma glucose (*p* < 0.001) and i resistance (HOMA-IR) (*p* < 0.001) in lactovegetarians group. No difference in beta-cell function between the two groups (*p* = 0.062).
Kuo et al. [34]	2004	Taiwan	36 healthy participants (omnivore—55.7 ± 3.7; vegetarians—58.6 ± 3.6 years old)	19 vegetarians 17 omnivores	-	Significantly lower levels of steady-state plasma glucose (SSPG) (*p* < 0.001), fasting insulin (*p* = 0.004), HOMA-IR (*p* = 0.002), HOMA %S (*p* = 0.018).
Kahleova et al. [35]	2011	Czech Republic	74 participants with type 2 diabetes (experimental group—54.6 ± 7.8, control group—57.7 ± 4.9 years old)	(1) experimental group (*n* = 37; vegetarian diet) (2) the control group (*n* = 37; conventional diet)	24 weeks	Reduced diabetes medication in the experimental group (43% participants; *p* < 0.001). Decreased body weight (*p* = 0.001) and visceral and subcutaneous fat in the experimental group (*p* = 0.007 and *p* = 0.02, respectively). Increased insulin sensitivity (*p* = 0.04) and plasma adiponectin (*p* = 0.02) in the experimental group.
Valachovicová et al. [36]	2006	Slovak Republic	202 participant (healthy adult subjects (age range 19–64 years; BMI 18.6–25.0 kg/m^2^)	(1) a vegetarian group (95 long-term lacto-ovo-vegetarians) (2) a non-vegetarian control group (107 participants on a traditional western diet)	-	Significantly lower glucose (*p* < 0.001), insulin concentrations (*p* < 0.01) and IR (HOMA) (*p* < 0.01) in the vegetarian group. Significantly higher intake of whole grain products, pulses, products from oat and barley (*p* < 0.001) in the vegetarians group.
Chiang et al. [37]	2013	Taiwan	706 female participants (age 56.4 ± 8.4 years old, overall healthy)	391 vegetarians (~80% lacto-ovo-vegetarians) 315 non-vegetarians	-	Significantly lower body mass index (*p* < 0.001), waist circumference (*p* < 0.001), lower total cholesterol (*p* < 0.001), LDL cholesterol (LDL-C) (*p* < 0.001), glucose (*p* < 0.001), insulin (*p* < 0.001), HOMA-IR (*p* < 0.001) and the risks for the MetS (*p* = 0.006).
Vučić Lovrenčić et al. [38]	2020	Croatia	76 participants (healthy non-obese adult, age- and gender matched; BMI < 30 kg/m^2^; 18–60 years old)	Vegetarians (*n* = 40) Omnivore (*n* = 36)	-	Significantly higher levels of adiponectin in female (*p* = 0.03) and the HOMA2-%B in vegetarians group than omnivore controls (*p* = 0.04). No differences in HOMA2-IRI, inflammatory and metabolic biomarkers.
Ellsworth et al. [39]	2016	USA	325 participants (subjects with diagnosed type-2 diabetes, CAD or significant risk factors; average age was 60.3 years (range 40.7–79.8)—intensive lifestyle and 61.5 years (range 33.9–86.2) in moderate lifestyle)	(1) intensive non-randomised program with a strict vegetarian diet (*n* = 90 participants, 90 matched controls) (2) moderate randomised trial following a Mediterranean-style diet (*n* = 89 subjects, 58 controls)	1 year	Decrease in weight loss (−8.9% (95% CI, −10.3 to −7.4), intensive programme; −2.8% (95% CI, −3.8 to −1.9), moderate programme; adjusted *p* < 0.001) and the LPIR score (−13.3% (95% CI, −18.2 to −8.3), intensive; −8.8% (95% CI, −12.9 to −4.7), moderate; adjusted *p* < 0.01) in both intervention with an advantage in the vegetarian diet.
Garousi et al. [40]	2021	Iran	75 participants (overweight/obese adults with NAFLD, aged between 20 and 55 years)	(1) lacto-ovo-vegetarian diet (LOV-D) (*n* = 37) (2) a standard weight-loss diet (SWL-D) (*n* = 38)	3 months	Decreased levels of alanine aminotransferase (ALT) (*p* < 0.001), body weight (*p* < 0.001), waist circumference (*p* < 0.001), BMI (*p* < 0.001), fasting blood sugar (*p* < 0.001), insulin (*p* < 0.001), HOMA-IR (*p* < 0.001), triacylglycerol (TG) (*p* = 0.001), cholesterol (*p* < 0.001), LDL cholesterol (*p* < 0.001),and systolic blood pressure (*p* = 0.001) in LOV-D group.
Chen et al. [41]	2018	The Netherlands	6798 participants (age 62.0 ± 7.8)	(1) 6514 participants for plant-based diet with insulin resistance (2) 5768 participants for a plant-based diet with prediabetes risk (3) 6770 participants for a plant-based diet with T2D risk	-	Higher score on the plant-based dietary index was associated with lower insulin resistance (per 10 units higher score: β = −0.09; 95% CI: −0.10; −0.08), lower prediabetes risk (HR = 0.89; 95% CI: 0.81; 0.98) and lower T2D risk (HR = 0.82 (0.73; 0.92)).

**Table 3:** The influence of a vegan diet on insulin resistance.

Study	Year	Country	Cohort	Analysed Groups	Time of Intervention	Results
Kahleova et al. [54]	2018	USA	75 participants (healthy overweight or obese adult men and women, BMI between 28 and 40 kg/m^2^, age 53.2 ± 12.6 years old)	(1) a plant-based diet (*n* = 38) (2) a control diet (current participant’s diet) (*n* = 37)	16 weeks	Significant reductions in body weight (−6.5 kg; *p* < 0.001), fat mass (−4.3 kg; *p* < 0.001) and HOMA-IR (−1.0; *p* = 0.004) in the vegan group.
Barnard et al. [55]	2021	USA	62 participants (healthy, overweight adults, BMI between 28 and 40 kg/m^2^, group 1—56.6 ± 10.9 years old, group 2—58.3 ± 8.4 years old)	(1) group on the Mediterranean diet (2) group on a low-fat vegan diet	16 weeks	Decreased weight (−6.0 kg; *p* < 0.001) and HOMA-IR (−0.7; *p* = 0.21) in vegan group. Increased oral glucose insulin sensitivity (OGIS) (+35.8 mL/min/m^2^; *p* = 0.003) in vegan group. No significant change in the Mediterranean diet group.
Kahleova et al. [56]	2018	USA	75 participants (healthy, overweight adults with a BMI between 28 and 40 kg/m^2^, age 53.2 ± 12.6 years old)	(1) plant-based high-carbohydrate, low-fat (vegan) diet (*n* = 38) (2) control group (current participant’s diet) (*n* = 37)	16 weeks	Significant reduction in body weight (−6.5 kg; *p* < 0.001), fat mass (−4.3 kg; *p* < 0.001) and HOMA-IR (−1.0; *p* = 0.004) in the vegan group.
Kahleova et al. [57]	2020	USA	168 participants (overweight, but otherwise healthy adult men and women with a BMI between 28 and 40 kg/m^2^; vegan group—52.9 ± 11.7 years old, control group—57.5 ± 10.2 years old)	(1) vegan group (*n* = 84) (2) control group (current participant’s diet) (*n* = 84)	16 weeks	Decreased body weight (−5.9 kg; *p* < 0.001), fat mass (−3.9 kg; *p* < 0.001) and visceral fat (−240 cm^3^; *p* < 0.001) in the vegan group. Increased PREDIcted M, insulin sensitivity index (PREDIM) in the vegan group (+0.83; *p* < 0.001). Significant changes in gut microbiota (*p* < 0.001) due to the low-fat vegan diet.
Kahleova et al. [58]	2019	USA	75 participants (healthy, overweight adults with a BMI between 28 and 40 kg/m^2^, age 53.2 ± 12.6 years old)	(1) low-fat vegan diet (*n* = 38) (2) control diet (current participant’s diet) (*n* = 37)	16 weeks	Decreased intakes of C18:0 (*p* = 0.004) and CLA-trans-10-cis12 (*p* = 0.002) in the vegan group. Increased intake of C18:2 (*p* = 0.002) and C18:3 (*p* = 0.006). Changes in the consumption of fatty acids have caused a decrease in HOMA-IR (*p* = 0.02) in the vegan group. The main fatty acids associated with changes in fasting insulin secretion were C12:0 (*p* = 0.03) and TRANS 16:1 (*p* = 0.02).
Kahleova et al. [59]	2018	USA	75 participants (healthy, overweight adults with a BMI between 28 and 40 kg/m^2^, age 53.2 ± 12.6 years old)	(1) vegan group (low-fat plant-based diet) (*n* = 38) (2) control group (current participant’s diet) (*n* = 37)	16 weeks	Decreased significantly HOMA-IR in the vegan group (−1.0; *p* = 0.004). Changes in HOMA-IR correlated positively with changes in body mass index (*p* = 0.009) and visceral fat volume (*p* = 0.001).
Kahleova et al. [60]	2021	USA	244 healthy participants (intervention group—52.6 ± 14.7 years old, control group—54.3 ± 9.9 years old)	(1) intervention group (vegan) (*n* = 122) (2) control group (current participant’s diet)(*n* = 122)	16 weeks	Reduction in Potential Renal Acid Load (PRAL) (−24.7 mEq/day; *p* < 0.001) and Net Endogenous Acid Production (NEAP) (−23.8 mEq/day; *p* < 0.001), body weight (−5.9 kg; *p* < 0.001) and HOMA-IR (*p* = 0.008) in vegan group. Increased PREDIM in the vegan group (*p* < 0.001).
Kahleova et al. [61]	2020	USA	244 healthy participants (BMI between 28 and 40 kg/m^2^, age 25 to 75 years)	(1) intervention group(low-fat vegan diet) (*n* = 122) (2) control group (current participant’s diet) (*n* = 122)	16 weeks	Decreased body weight (−5.9 kg; *p* < 0.001), HOMA (−1.3; *p* < 0.001), hepatocellular lipid levels (−34.4%; *p* = 0.002) and intramyocellular lipid levels (−10.4%; *p* = 0.03) in the intervention group. Increased thermic effect of food (+14.1%; *p* < 0.001) and PREDIM (+0.9; *p* < 0.001) in the intervention group. No significant changes in the control group.
Barnard et al. [62]	2005	USA	64 participants (overweight or obese, postmenopausal women; mean age for intervention group—57.4 y, for control group—55.6 y)	(1) intervention group (low-fat, vegan diet) (2) control group (control diet based on National Cholesterol Education Program guidelines)	14 weeks	Decreased body weight (−5.8 ± 3.2 kg in the intervention group; −3.8 ± 2.8 kg in the control group; *p* = 0.012). Increased index of insulin sensitivity (from 4.6 ± 2.9 to 5.7 ± 3.9; *p* = 0.017) in the intervention group.
Śliż et al. [63]	2021	Poland	98 participants (healthy Polish males, athletes, aged 20–39 years)	(1) vegan group (VEG; *n* = 44) (2) omnivore group (OMN; *n* = 54)	-	Higher intake of carbohydrate (*p* < 0.01), unsaturated fatty acids (*p* < 0.01) in the VEG group. Lover intake of protein (*p* < 0.01), fat (*p* < 0.01), saturated fatty acids (*p* < 0.01) and EPA + DHA (*p* < 0.01) in the VEG group. Significantly better outcomes in *n*-6/n-3 fatty acid ratio (6.5% ± 2.3% vs. 5.0% ± 2.1%; *p* < 0.01) insulin sensitivity (HOMA-IR), C-peptide and total blood cholesterol levels (*p* < 0.01) in the VEG group.
